# Discovery of LAH-1 as potent c-Met inhibitor for the treatment of non-small cell lung cancer

**DOI:** 10.1080/14756366.2023.2286435

**Published:** 2023-12-11

**Authors:** Lijie Sima, Zhongyuan Wang, Ling Yu, Youli Hou, Dongsheng Zhao, Bilan Luo, Weike Liao, Xinfu Liu

**Affiliations:** a Department of Hematology and Oncology, The Affiliated Shaoyang Hospital, Hengyang Medical School, University of South China (Shaoyang Central Hospital), Shaoyang, China; b Department of Pharmacy, Guizhou Provincial People’s Hospital, Guiyang, China; c Department of Pharmacy, Guiyang Healthcare Vocational University, Guiyang, China; d Guizhou Provincial Engineering Technology Research Center for Chemical Drug R&D, Guizhou Medical University, Guiyang, China

**Keywords:** c-Met, LAH-1, antitumor activity, NSCLC

## Abstract

Dysregulated HGF/c-Met pathway has been implicated in multiple human cancers and has become an attractive target for cancer intervention. Herein, we report the discovery of *N*-(3-fluoro-4-((2-(3-hydroxyazetidine-1-carboxamido)pyridin-4-yl)oxy)phenyl)-1-(4-fluorophenyl)-4-methyl-6-oxo-1,6-dihydropyridazine-3-carboxamide (**LAH-1**), which demonstrated nanomolar MET kinase activity as well as desirable antiproliferative activity, especially against EBC-1 cells. Mechanism studies confirmed the effects of **LAH-1** on modulation of HGF/c-Met pathway, induction of cell apoptosis, inhibition on colony formation as well as cell migration and invasion. In addition, **LAH-1** also showed desirable *in vitro* ADME properties as well as acceptable *in vivo* PK parameters. The design, synthesis, and characterisation of **LAH-1** are described herein.

## Introduction

Nowadays, cancers are still the major contributor to disease burden. With approximately 2.2 million new cases and 1.8 million deaths per year, lung cancer has become the most common malignant disease worldwide. Based on histologic types, lung cancer can be divided into two subtypes: non-small cell lung cancer (NSCLC, 85%) and small cell lung carcinoma (SCLC, 15%). Owing to the discovery of druggable genetic alterations and mutations such as EGFR, ROS1, ALK, RET, FGFR1, MET Exon 14 and NTRK, the management of NSCLC has been altered dramatically. These biomarker-driven targeted therapies are now the standard of care for molecularly defined populations^1–3^.

Among the targets for cancer intervention, c-Met and its ligand HGF have drawn considerable attention^4^^,^^5^. Met, encoded by the MET proto-oncogene, is a subfamily of receptor tyrosine kinases (RTKs) that also includes RON and SEA. The only identified high affinity ligand for c-Met is hepatocyte growth factor (HGF), which is secreted by fibroblast cells. Genetic studies show that both c-Met and HGF play essential roles in normal embryonic development and organogenesis^6^. However, in most malignancies including lung, brain, gastric, colorectal, head and neck cancers, the HGF/c-Met pathway is dysregulated via multiple mechanisms, such as HGF binding, gene rearrangement, amplification, or mutation, thus promoting the activation of multiple downstream signalling pathways^7^. Moreover, c-Met is also reported to interact with other protein or enzymes, which include a6b4 integrin, CD44, FAS, RON and EGFRs, most of which show synergistic effects on cancer progression and the development of acquired resistance to approved therapies^8^. Giving these facts, targeting c-Met has become a promising strategy for cancer intervention.

**Figure 1.**
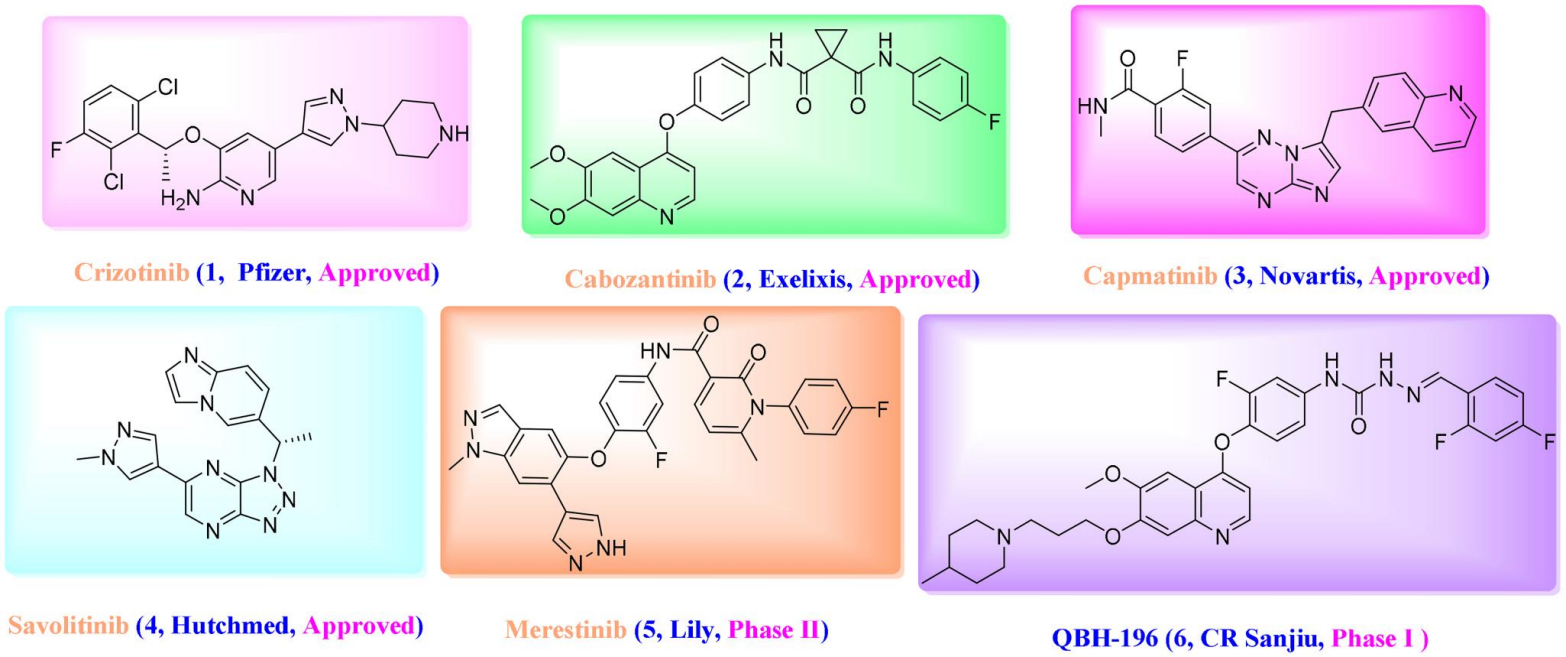
Representative structures of reported c-Met inhibitors.

Considering the effects of c-Met on cancer progression, several small molecule inhibitors have been pursued by pharmaceutical companies and researchers^9–11^, these include Crizotinib (Launched, 2011), Cabozantinib (Launched, 2012), Capmatinib (Launched, 2020), Savolitinib (Launched, 2021), Merestinib (Phase II) and so on (Figure 1)^12–14^. Despite the progress that has been made, all the patients would inevitably acquire resistance to these drugs, similar to what will happen when other TKI treatments are used. Therefore, novel inhibitors with desirable c-Met inhibition are still needed.

**Figure 2.**
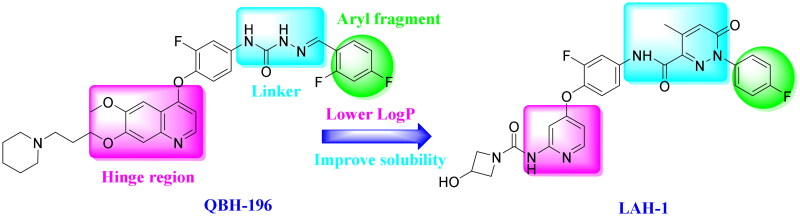
The design strategy of **LAH-1** based on QBH-196.

Among the inhibitors that have been reported, a series of quinolines-based derivatives have attracted our attention^15–17^. QBH-196 (**6**), a novel 4-phenoxy-6,7-disubstituted quinolines that shows excellent c-Met inhibition as well as desirable activity in a human gastric cancer xenograft model, is now in phase I clinical trials. However, its high lipophilicity as well as poor solubility needs to be improved. In our effort to design novel c-Met inhibitors, the quinoline ring of QBH-196 was replaced with pyridine, and urea moiety with terminal aliphatic amine, preferably azetidin-3-ol^17^, was introduced into the 2-position of pyridine, preserving the key interactions with the hinge region as well as improving the druggability. Besides, the semicarbazone linker, which was responsible for addition hydrogen bonds with c-Met kinase, was also replaced with pyridazinone moiety based on our previous SAR studies^15^. Moreover, the most favourable 4-flurophenyl group from Cabozantinib as well as Merestinib was preserved to extend into the hydrophobic pocket, this led to the discovery of **LAH-1** (Figure 2).

In this paper, we communicate medicinal chemistry lead optimisation of QBH-196 leading to the discovery of *N*-(3-fluoro-4-((2–(3-hydroxyazetidine-1-carboxamido)pyridin-4-yl)oxy)phenyl)-1–(4-fluorophenyl)-4-methyl-6-oxo-1,6-dihydropyridazine-3-carboxamide (**LAH-1**). The effects of **LAH-1** on inhibiting the HGF/MET pathway on tumour progression, and its potential application in the treatment of NSCLC are described herein.

## Results and discussion

### Chemistry

**Scheme 1.**
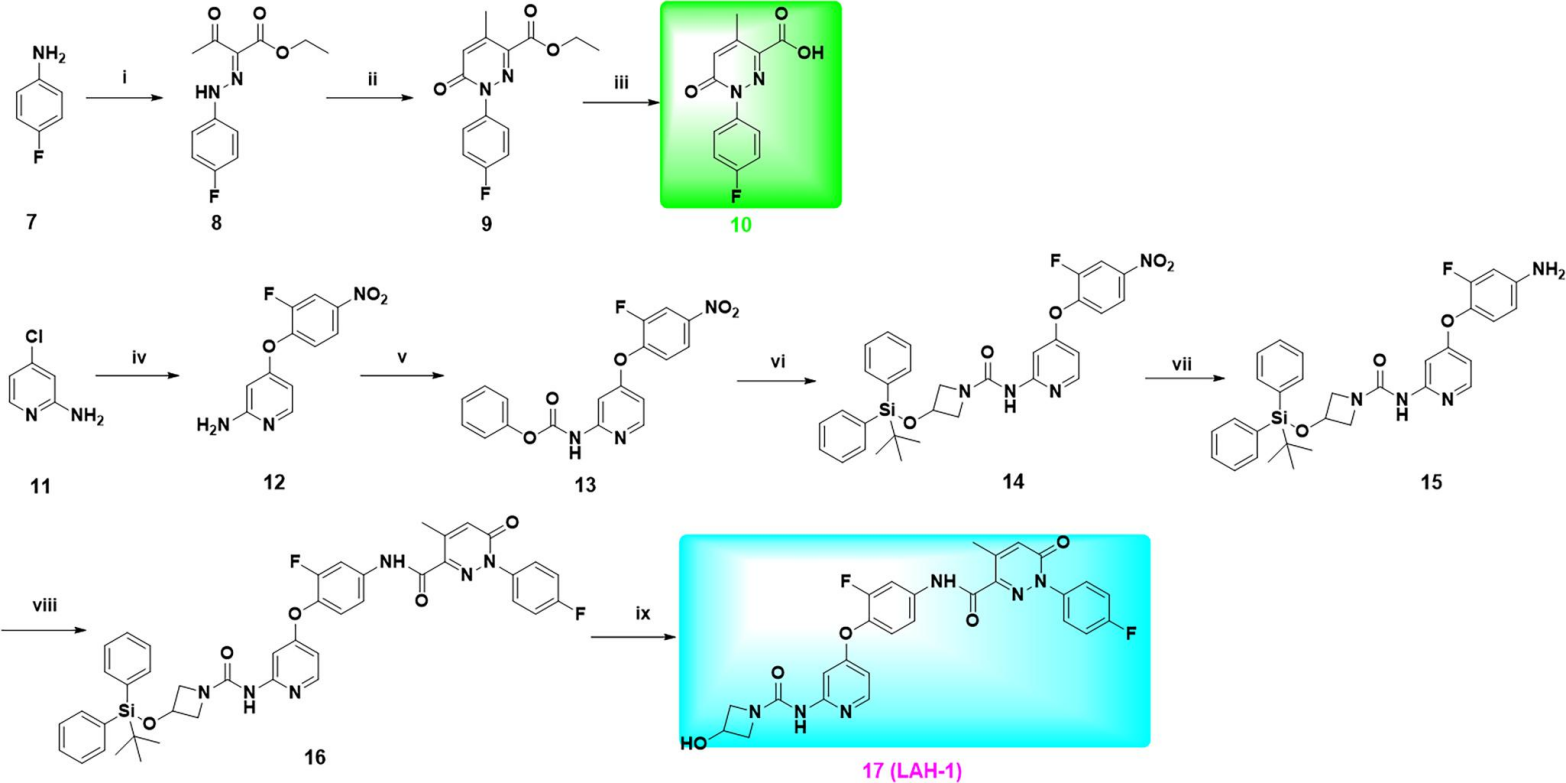
Reagents and conditions: (i) a) NaNO_2_, 15% HCl, H_2_O, 0 °C, 30 min; b) Ethyl acetylacetate, C_2_H_5_COONa, EtOH/H_2_O, 0–25 °C, 2 h; (ii) Ph_3_P = CHCOOC_2_H_5,_ PhMe, 110 °C, 12 h; (iii) 10% NaOH, EtOH/THF, 50 °C, 4 h; (iv) 2-fluoro-4-nitrophenol, Pyridine, PhCl, 135 °C, 72 h; (v) Phenyl chloroformate, Pyridine, CH_2_Cl_2_, 0 °C, 30 min; (vi) 3-((tert-butyldiphenylsilyl)oxy)azetidine Et_3_N, THF, 70 °C, 4 h; (vii) Pd/C, H_2_, EtOH, r.t, 5 h; (viii) **10**, HATU, DIPEA, DMF, r.t, 12 h; (ix) TBAF, THF, 0 °C to r.t, 30 min.

As shown in Scheme 1, a nine-step synthetic sequence was used for the preparation of targeted compound **LAH-1**. Briefly, the diazotised salt of 4-fluroaniline was reacted with ethyl acetoacetate in the presence of sodium acetate to afford intermediate **8**, further cyclisation with (carbethoxymethylene)triphenylphosphorane in refluxing toluene afforded intermediate **9**, which was then hydrolysed to corresponding acid **10** using 10% sodium hydroxide. On the other hand, 4-chloropyridin-2-amine was substituted with 2-fluoro-4-nitrophenol in the presence of pyridine at 135 °C to give intermediate **12**, further substitution with phenyl chloroformate and subsequent ammonolysis afforded intermediate **14**. The intermediate **14** could be easily converted into amine by catalytic hydrogenation under room temperature, subsequent condensation with carboxylic acid **10** yield intermediate **16**, which was then deprotected by TBFA in THF to give the desired target compound **LAH-1**.

### Biological evaluation

#### LAH-1 is a potent, selective c-Met inhibitor with nanomolar biochemical potency

**Figure 3.**
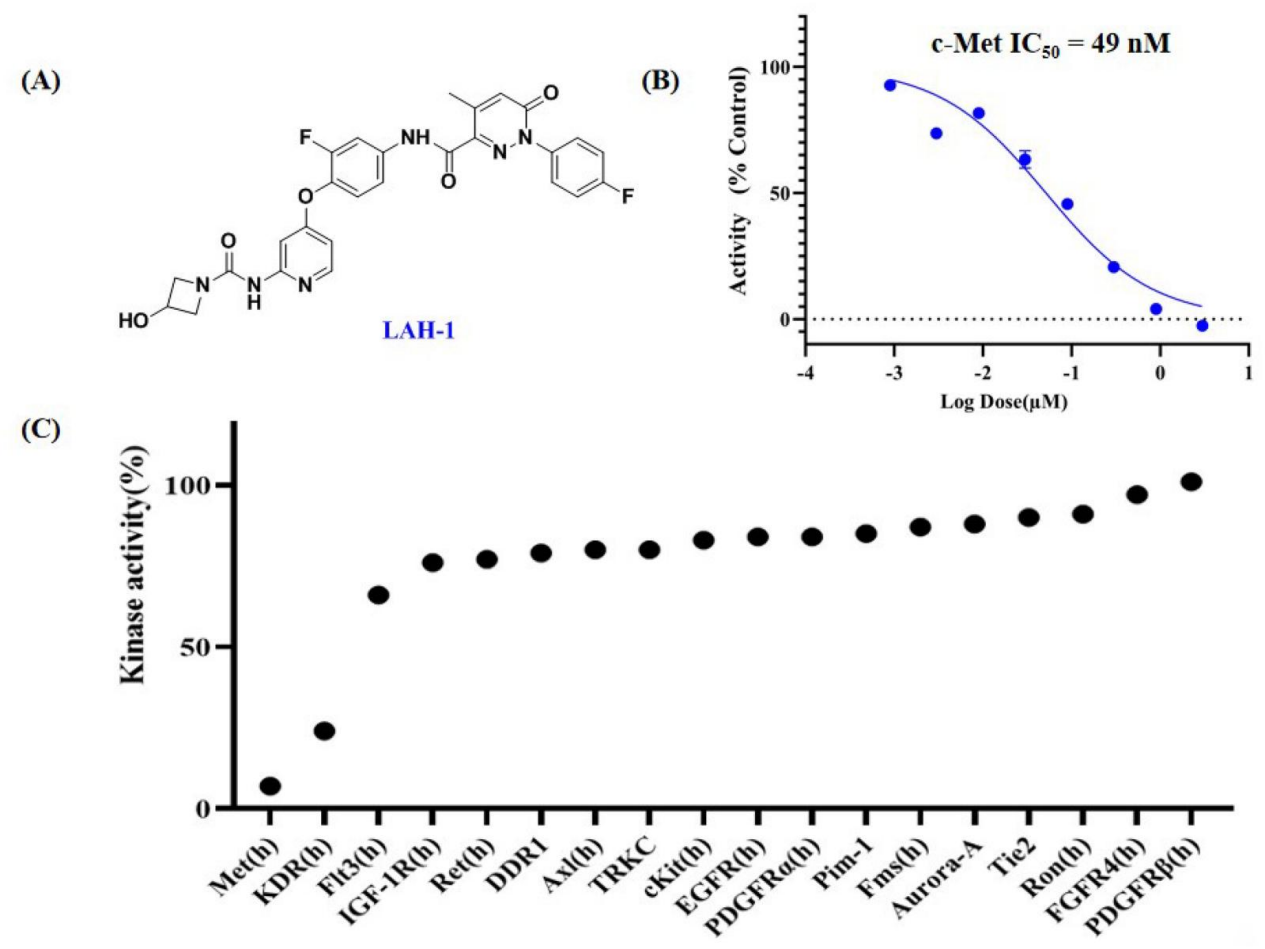
**(A).** The chemical structure of **LAH-1**; **(B)**. The IC_50_ determination of **LAH-1** against c-Met enzyme *in vitro*. **(C)**. Kinase profile of **LAH-1** against a panel of 18 kinases at 300 nM.

To evaluate the inhibitory activity of **LAH-1** against c-Met, the mobility shift assay (MSA) with ATP concentration at Km was utilised with Cabozantinib as positive control. As shown in Figure 3, **LAH-1** exhibited high potency against c-Met, with IC_50_ value of 49 nM, which was comparable with that of Cabozantinib (IC_50_ = 31 nM). Considering specificity as an important issue in kinase inhibitors, we further accessed the enzymatic activities of **LAH-1** against a panel of 18 diversified kinases at the concentration of 300 nM. As indicated in Figure 3, **LAH-1** showed great selectivity over the other 16 kinases that we evaluated, including highly homologous kinases RON and Axl. Notably, **LAH-1** also showed inhibition on KDR. Overall, these data indicated that **LAH-1** was a potent, selective c-Met inhibitor.

#### LAH-1 selective suppressed the viability of c-Met driven cancer cell proliferation

**Figure 4.**
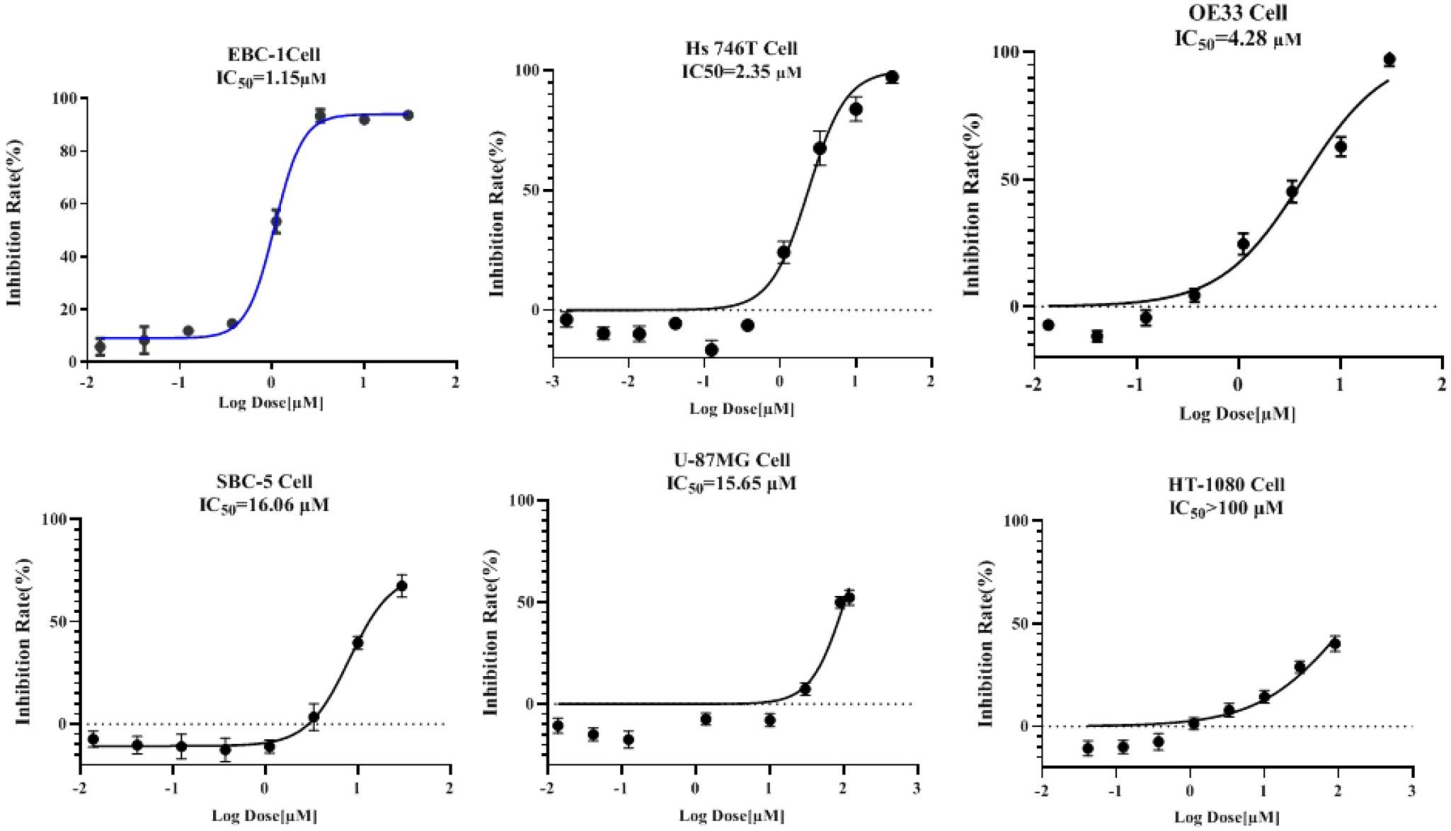
IC_50_ determination of **LAH-1** against 5 c-Met addicted cancer cell lines and 1 non-addicted cell line. IC_50_ values were determined after exposure of cells to derivatives for 72 h, and data are expressed as the mean ± SD of two independent experiments.

Considering increased c-Met activity has been implicated in multiple tumour cells, we evaluated the half maximal inhibitory concentration (IC_50_) of **LAH-1** in several cancer cell lines which harbour different levels of Met genetic alteration, these include human lung squamous cell carcinoma EBC-1, human gastric cancer line Hs746T, human oesophageal adenocarcinoma cell line OE33, human small-cell lung cancer SBC-5, and human glioblastoma cell U87MG. As shown in Figure 4, **LAH-1** was efficacious against these five cell lines, with IC_50_ values of 1.15 μM, 2.35 μM, 4.28 μM, 16.06 μM, 15.65 μM, respectively. In contrast to cell lines with low expression or activation of c-Met, **LAH-1** had a minimal effect on human fibrosarcoma cell line HT-1080, which further validated its target at the cellular level. Moreover, **LAH-1** also exerted little antiproliferative inhibition against human lung fibroblast cell line WI-38, with only 34.5% inhibition at the concentration of 100 μM.

#### LAH-1 dose-dependent inhibited c-Met phosphorylation and its downstream signalling in EBC-1 cells

**Figure 5.**
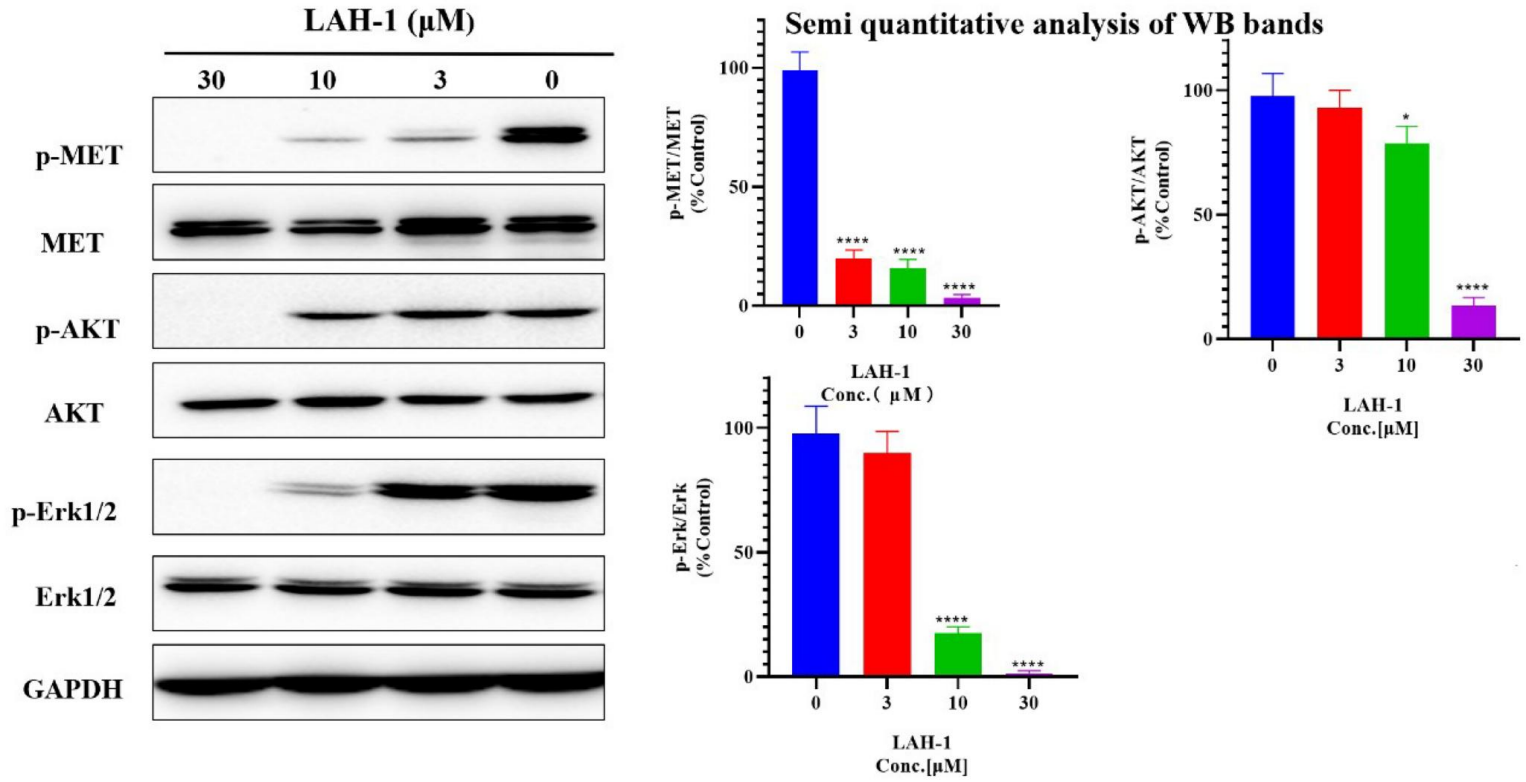
Western blot analyses of EBC-1 cells. Cells were treated with **LAH-1** at the indicated concentrations for 2 h, and GADPH was used as a loading control. Each experiment was done in double, and representative images are shown, *p < 0.05, **p < 0.01, ***p < 0.001, ****p < 0.0001 as compared with control.

Based on the sensitivity of **LAH-1** against six cancer cell lines, we then conducted western blot assays to confirm the effect of **LAH-1** on c-Met inhibition in cancer cells, and human non-small lung cancer (NSCLC) cell EBC-1 which harbours amplified Met gene was used. As indicated by Figure 5, a dose-dependent decrease in phosphorylation level of c-Met, Akt, and Erk 1/2 was observed in EBC-1 cells, which suggest the inhibition of c-Met as well as PI3K and Ras signalling axis.

#### LAH-1 dose-dependent induced cell apoptosis

**Figure 6.**
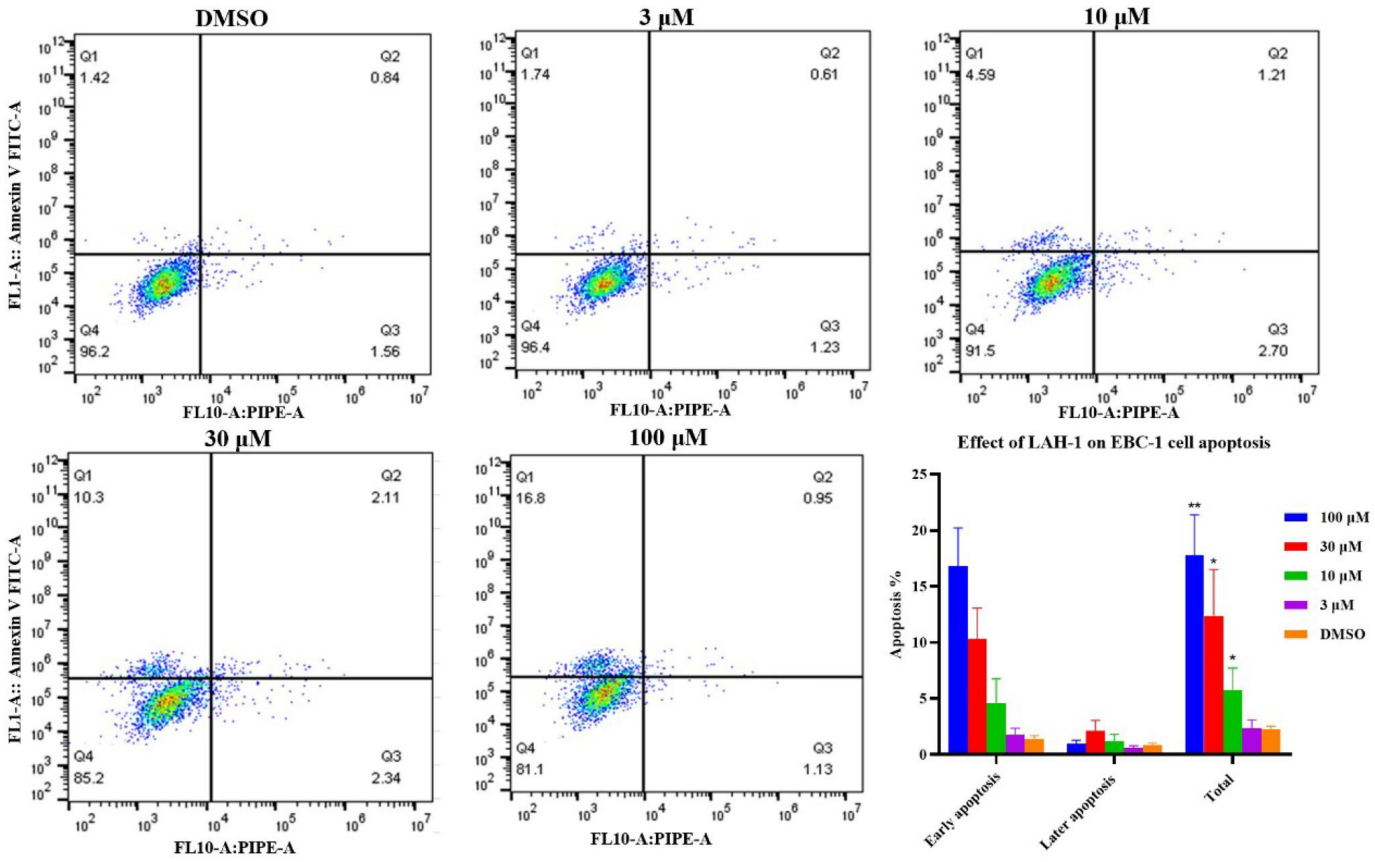
Quantification of apoptotic EBC-1 cells by Annexin V-FITC/PI dual staining, the data shown are the mean ± SD from two independent experiments. Q1: Early apoptotic cells, Q2: Late apoptotic cells. Q3: Necrotic cells, Q4: Living cells. *p < 0.05, **p < 0.01 as compared with control.

To determine whether the observed cell death was due to physiological apoptosis, Annexin V FITC/PI dual staining assay was conducted. EBC-1cells were treated with **LAH-1** at different concentrations for 24 h to examine the effect on apoptosis. As can be seen from Figure 6, **LAH-1** showed significant induction of apoptosis against EBC-1cells, with early apoptosis from 1.42% to 16.8% compared to the untreated group.

#### LAH-1 inhibited the colony formation, migration, and invasion in c-Met dependent cells

**Figure 7.**
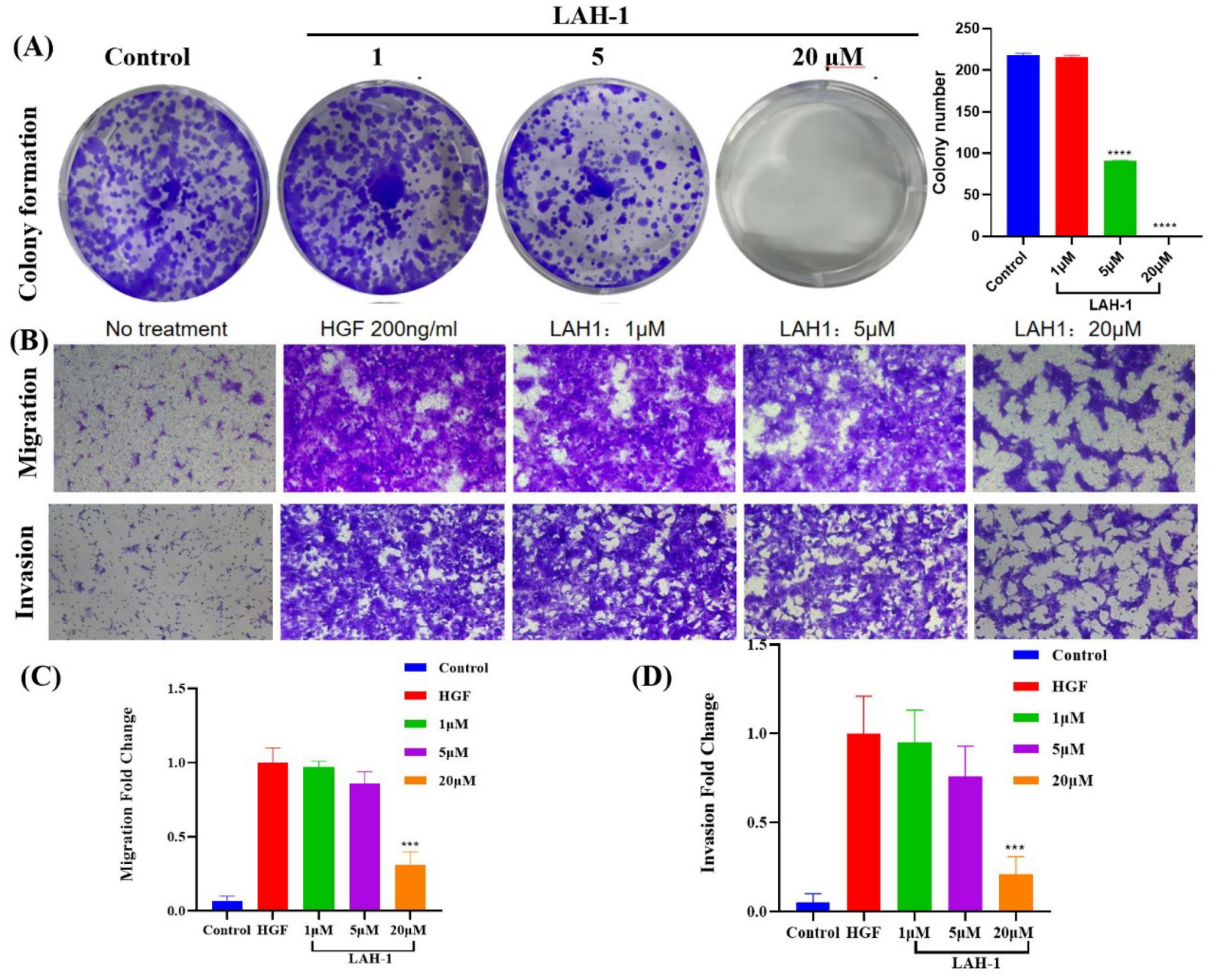
**(A).** EBC-1 cells were treated with different concentration of **LAH-1**. Cell plates were stained with crystal violet and imaged. Two independent experiments were carried out, and a representative plate is shown. **(B)**. **LAH-1** suppressed HGF-induced NCI-H441 cell invasion and migration. Representative images are shown. The relative migration (C) and invasion (D) fold change were plotted. The data shown are the mean from two independent experiments. *p < 0.05, **p < 0.01, ***p < 0.001, ****p < 0.0001 as compared with control.

To investigate the effect of **LAH-1** on colony formation ability, the EBC-1 cells which harbouring Met amplification were treated with different concentrations of **LAH-1**. As shown in Figure 7, colony formation was significantly inhibited after 14 days treatment, especially at the concentration of 20 μM. Considering the important role of HGF/c-Met signalling in promoting cancer cell migration and invasion, the NCI-H441 cells with elevated expression level of c-Met was used. After treatment with HGF in the presence of LAH-1, the migratory and invasive abilities of NCI-H441 cells were determined. As shown in Figure 7, **LAH-1** dose dependent suppressed the migration and invasion of HGF-induced NCI-H441 cells.

#### LAH-1 showed acceptable in vitro and in vivo ADME properties

Having characterised **LAH-1** as a potent c-Met inhibitor, the ADME properties and PK parameters were further determined. As depicted in Table 1, **LAH-1** demonstrated low intrinsic clearance in both SD rat and human liver microsomes, with clearance lower than 8.6 μL/min/mg and half-life higher than 125 min. Additionally, Egg-PAMPA (Parallel artificial membrane permeability assay) confirmed **LAH-1** as membrane permeable, with permeability rates (P_e_) of 3.4 nm/s. Supplement to these data, **LAH-1** was further screened for its inhibition activity on five major CYP450 enzymes (CYP1A2, CYP2D6, CYP2C9, CYP2C19, and CYP3A4). As shown in Table 1, **LAH-1** showed little or minimal inhibition on five CYP450 enzymes at concentration of 10 μM, despite 50.7% inhibition for CYP2C9 was observed. Next, **LAH-1** was further progressed into *in vivo* PK studies. When intravenously administered at a dose of 5 mg/kg in SD rats, moderate plasma clearance was found. When orally administered at a dose of 20 mg/kg, **LAH-1** was quickly absorbed, with T_max_ of 0.33 h and AUC0-∞ of 8210 ng•h/mL. The oral bioavailability of **LAH-1** was determined to be 12.2%.

**Table 1.** *In Vitro* ADME and *in vivo* PK parameters for **LAH-1**.

**Metabolic stability** ^a^						
Species		T_1/2_(min)		CL _int_(μL/min/mg)	Remaining (T = 60min)	
RLM		> 125		< 8.6	81.2%	
HLM		> 125		< 8.6	88.7%	
**Permeability assay** ^b^						
Egg-PAMPA		Mean Pe (nm/s)		Incubation Time (h)	Permeability	
–		3.4		4	Moderate	
**CYP450 inhibition** ^c^						
isozyme		CYP1A2	CYP2D6	CYP2C9	CYP2C19	CYP3A4
% inhibition		12.8	21.5	50.7	15.6	32.2
**Pharmacokinetics** ^d^						
	Tmax (h)	C_max_(μg/mL)	AUC 0-∞ (ng•h/mL)	T_1/2_ (h)	CL (mL/min/kg)	F%
iv	–	14.9	16767	1.6	19.2	–
po	0.33	4.46	8210	2.2	–	12.2

^a^ Testosterone and Diclofenac were used at positive control.

^b^ The permeability assay was Performed at 10 μM concentration.

^c^ Performed at 10 μM concentration. α-Naphthoflavone (CYP1A2), Squinidine (CYP2D6), (+)-N-3-benzylnirvanol (CYP2C19), Quinidine (CYP2D6), and ketoconazole (CYP3A4) were used as the positive controls.

^d^ Determined from intravenous or PO dosing to male SD rats at a dose of 5 mg/kg and 20 mg/kg, respectively (*n* = 3).

### Molecular modelling and dynamic simulation

**Figure 8.**
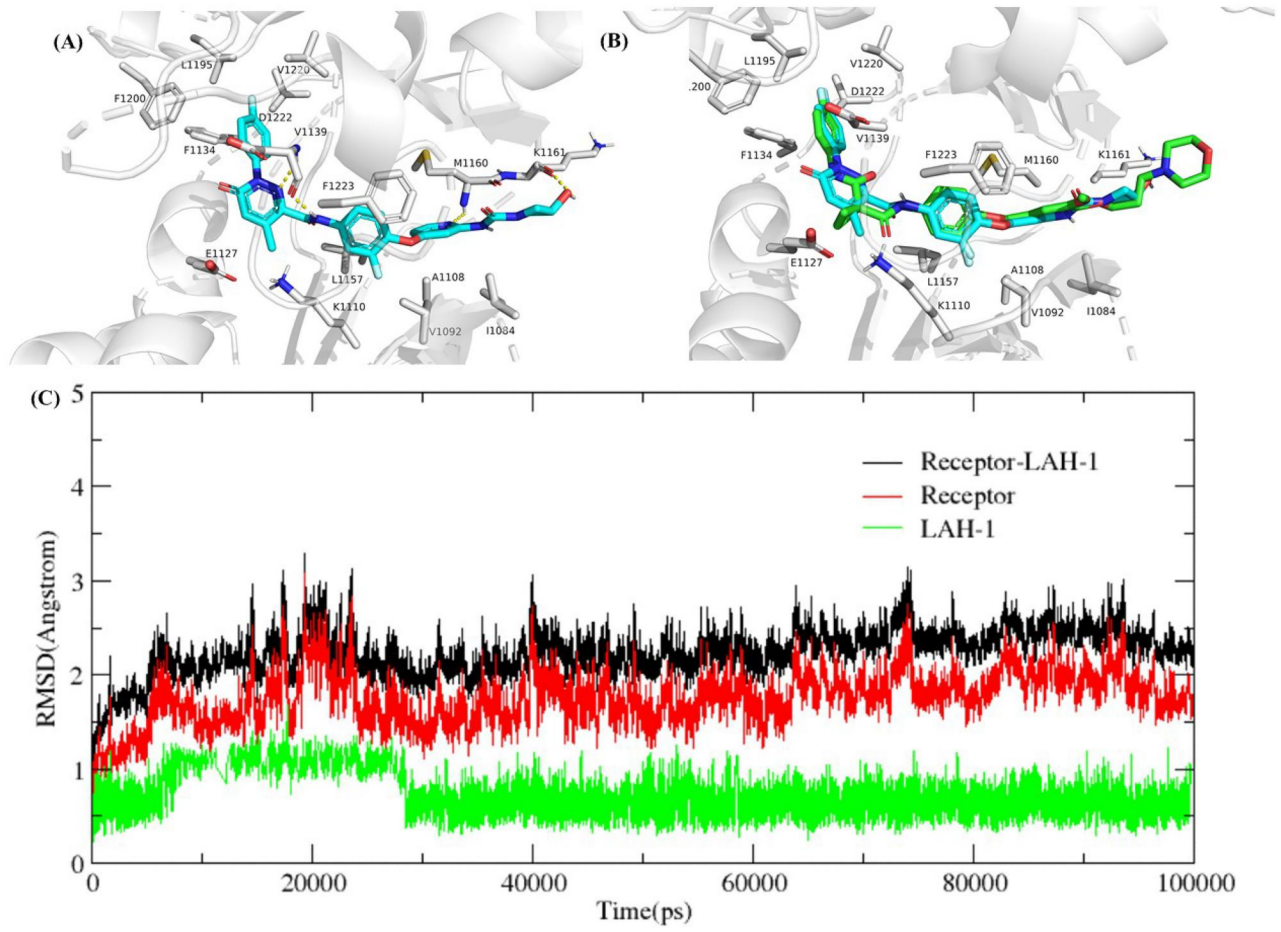
(A) Predicted binding mode of **LAH-1** to c-Met (3LQ8). Hydrogen bonds are indicated by yellow dashed lines. Images are generated using PyMol. (B) The overlay of compound **LAH-1** with Foretinib in c-Met. (C) Dynamics of **LAH-1** bound to c-Met (3LQ8) during 100 ns simulation time.

To further elucidate the binding mode between **LAH-1** and c-Met, the structure of c-Met (PDB ID code: 3LQ8) was selected as the docking model. As expected, **LAH-1** adopted a similar binding mode of Foretinib. As shown in Figure 8, the nitrogen of pyridine and the oxygen of azetidin-3-ol form important hydrogen bonds with Met 1160 and Lys 1161 in the hinge region, respectively. Another typical hydrogen bond between the dihydropyridazine-3-carboxamide and Asp 1222 can also be observed. Additionally, the fluoro substituted phenyl forms π − π stacking interaction with Phe 1223, which support the excellent kinase activity for c-Met. To access the binding stability of **LAH-1**/c-Met adducts, we subsequently conducted a 100 ns long molecular dynamic simulation, and the RMSD (Root Means Square Deviation) was calculated. As can be seen in Figure 8, the RMSD did not fluctuate significantly, the RMSD value for the **LAH-1**/c-Met complex was less than 3 Å in the 100 ns simulation time.

## 3. Conclusion

Through molecular hybridisation, a pyridine-based derivative containing pyridazine-3-carboxamide moiety (**LAH-1**) was designed and synthesised, this compound exhibited excellent c-Met kinase activity as well as selectivity against a panel of 18 kinases that we screened. Besides, it also showed desirable inhibition on five c-Met dependent neoplastic phenotypes of cancer cells. Mechanism studies revealed that **LAH-1** potent and sustained inhibited Met, Akt, and Erk phosphorylation, colony formation, as well as cell migration and invasion. Subsequent *in vitro* ADME evaluation confirmed that **LAH-1** was metabolic stable, cell permeable, and avoid of drug-drug interaction. Finally, the *in vivo* PK study was conducted and acceptable oral bioavailability (12.2%) was achieved in SD rats. Further optimisation work focused on **LAH-1** is in progress and will be disclosed in due course.

## Experimental section

### Chemistry

Unless otherwise specified, all the chemical reagents and solvents in this paper were of reagent grade and obtained from commercial sources without further purification. The detection of reactions was carried out by TLC using the silica Gel GF254 plates from Qingdao Ocean Chemicals. The purity of all the intermediates and target compound were analysed by HPLC (Agilent 1260 infinity), with a purity ≥ 95%. Melting points were determined on a Büchi Melting Point B-540 apparatus and were uncorrected. Flash chromatography was conducted using silica gel (200–300 mesh) from Qingdao Ocean Chemicals. Mass spectra (MS) were taken in ESI mode on Waters e2695.^1^H NMR and ^13^C NMR spectra were recorded on Bruker ARX-600 with tetramethylsilane (TMS) as an internal standard.

### General procedures

#### Procedure for preparation of intermediate 8

A solution of NaNO_2_ (0.45 g, 6.5 mmol) in water (6.5 ml) was added drop-wise to the mixture of 4-fluoroaniline (0.60 g, 5.38 mmol) and 10% HCl (14.40 mmol) in an ice bath. Upon the completion of the addition, the reaction mixture was stirred at 0 °C for 30 min, and then added into a mixture of ethyl acetyl acetate (0.70 g, 5.38 mmol), sodium acetate (1.33 g, 16.16 mmol), and EtOH/H_2_O (20 ml, 3:1) at 0 °C. The mixture was stirred for another 2 h and then filtered, washed with water, dried under vacuum to yield ethyl (*E*)-2-(2-(4-fluorophenyl)hydrazineylidene)-3-oxobutanoate (**8)** as a light yellow solid. Yield: 57.8%; HRMS(ESI) calculated for C_12_H_14_FN_2_O_3_ [M + H]^+^ m/z 253.0910, found: 253.0915.

#### Procedure for preparation of intermediate 9

Ethyl (triphenylphosphoranylidene)acetate (3.42 g, 9.83 mmol) and intermediate **8** (1.24 g, 4.92 mmol) were dissolved in dried toluene (30 ml) and stirred at 120 °C for 12 h. Upon completion, the solvent was removed under reduced pressure and the residue was triturated with petroleum ether. The solid obtained was collected by filtration and recrystallized from EtOH to afford pure ethyl 1-(4-fluorophenyl)-4-methyl-6-oxo-1,6-dihydropyridazine-3-carboxylate (**9**) as a white solid. Yield: 58.6%; HRMS(ESI) calculated for C_14_H_14_FN_2_O_3_ [M + H]^+^ m/z 277.0910, found: 277.0916.

#### Procedure for preparation of intermediate 10

To a solution of intermediate **9** (1.28 g, 4.65 mmol) in THF/EtOH (20 ml, 1:1) was added 10% NaOH (5 ml) at room temperature. The reaction mixture was then stirred at 50 °C for 5 h. After the reaction is complete, the solvent was removed under reduced pressure and the residue was poured into H_2_O (25 ml) and acidified with 6 N HCl to afford 1-(4-fluorophenyl)-4-methyl-6-oxo-1,6-dihydropyridazine-3-carboxylic acid (**10**) as white solid. Yield: 85.1%; HRMS(ESI) calculated for C_12_H_10_FN_2_O_3_ [M + H]^+^ m/z 249.0597, found: 249.0595.

#### Procedure for preparation of intermediate 12

To a suspension of 2-amino-4-chloropyridine (4 g, 31.11 mmol) and 2-fluoro-4-nitrophenol (5.86 g, 37.30 mmol) in chlorobenzene (70 ml) was added pyridine (1.25 ml, 15.56 mmol), the reaction mixture was stirred at 140 °C for 3 d and monitored by thin-layer chromatograph (TLC). Upon completion, the reactor was concentrated under reduced pressure and then dichloromethane (50 ml) was added, the residue was washed with H_2_O (30 ml × 2), saturated sodium carbonate (30 ml × 2) and then brine. The organic layer was dried over Na_2_SO_4_, filtered, and then concentrated to obtain a crude product, which was then purified by silica gel chromatography using PE/EA (1:1) to afford 4-(2-fluoro-4-nitrophenoxy)pyridin-2-amine (**12)** as a yellow solid. Yield: 90.3%; HRMS(ESI) calculated for C_11_H_9_FN_3_O_3_ [M + H]^+^ m/z 250.0550, found: 250.0556.

#### Procedure for preparation of intermediate 13

To a solution of 4-(2-fluoro-4-nitrophenoxy)-pyridin-2-amine (**12**) (7.5 g, 30.11 mmol) in CH_2_Cl_2_ (150 ml) was added pyridine (12.12 ml, 150.55 mmol), the reactor was cooled to 0 °C, and phenyl chloroformate (7.56 ml, 60.22 mmol) was added drop-wise. The mixture was then stirred at 0 °C for 30 min. Upon completion, the residue was washed with H_2_O (80 ml × 2), saturated sodium carbonate (60 ml × 2) and then brine. The obtained organic layer was dried over Na_2_SO_4_, filtered, concentrated to obtain crude product, which was purified by silica gel chromatography using a mixture of PE/EA (4/1) to afford pure phenyl (4-(2-fluoro-4-nitrophenoxy)pyridin-2-yl)carbamate (**13)** as a yellow solid. Yield: 90.0%; HRMS(ESI) calculated for C_18_H_13_FN_3_O_5_ [M + H]^+^ m/z 370.0761, found: 370.0765.

#### Procedure for preparation of intermediate 14

To a solution of phenyl (4-(2-fluoro-4-nitrophenoxy) pyridin-2-yl) carbamate (**13**) (10.00 g, 27.09 mmol) in THF (70 ml) was treated with Et_3_N (7.53 ml, 54.18 mmol) in one portion, 3-((tert-butyldiphenylsilyl)oxy) azetidine (10.11 g, 32.52 mmol) was then added. The mixture was stirred at 70 °C for 5 h. Upon completion, the reactor was concentrated under reduced pressure and then dichloromethane (50 ml) was added, the residue was washed consecutive with H_2_O (30 ml × 2), saturated sodium bicarbonate (30 ml × 2) and then brine. The organic layer was dried over Na_2_SO_4_, filtered, and concentrated under reduced pressure to afford 3-((tert-butyldiphenylsilyl)oxy)-*N*-(4-(2-fluoro-4-nitrophenoxy)pyridin-2-yl)azetidine-1-carboxamide (**14**) as a brown solid. Yield: 71.4%; HRMS(ESI) calculated for C_31_H_32_FN_4_O_5_Si [M + H]^+^ m/z 587.2048, found: 587.2052.

#### Procedure for preparation of intermediate 15

To a suspension of 3-((tert-butyldiphenylsilyl)oxy)-*N*-(4-(2-fluoro-4-nitrophenoxy)pyridin-2-yl)azetidine-1-carboxamide (**14**) in EtOH (120 ml) was added 10% palladium on carbon (21.61 mmol), the reactor was flushed with H_2_ and stirred at room temperature for 5 h. Upon completion, the reaction mixture was filtered, washed with EtOH (10 ml), concentrated to obtain crude product, which was purified by silica gel using CH_2_Cl_2_/EA (20:1) as fluent to afford *N*-(4-(4-amino-2-fluorophenoxy)pyridin-2-yl)-3-((tert-butyldiphenylsilyl)oxy)azetidine-1-carboxamide as a white solid. Yield: 85.2%; HRMS(ESI) calculated for C_31_H_34_FN_4_O_3_Si [M + H]^+^ m/z 557.2306, found: 557.2309.

#### Procedure for preparation of intermediate 16

To a suspension of intermediate **15** (0.2 g, 0.36 mmol), HATU (0.21 g, 0.56 mmol), DIPEA (0.094 g, 0.73 mmol) in DMF, 1-(4-fluorophenyl)-4-methyl-6-oxo-1,6-dihydropyridazine-3-carboxylic acid (0.13 g, 0.54 mmol) was added, the mixture was stirred at 25 °C for 12 h. The reaction mixture was poured into ice-water (20 ml), and the resulting precipitate was filtered, washed with H_2_O (5 ml) and dried under reduced pressure to afford product *N*-(4-((2–(3-((tert-butyldiphenylsilyl)oxy)azetidine-1-carboxamido)pyridin-4-yl)oxy)-3-fluorophenyl)-1–(4-fluorophenyl)-4-methyl-6-oxo-1,6-dihydropyridazine-3-carboxamide (**16**) as a yellow solid. Yield: 85.2%; HRMS(ESI) calculated for C_43_H_41_F_2_N_6_O_5_Si [M + H]^+^ m/z 787.2798, found: 787.2793.

#### Procedure for preparation of LAH-1

To a solution of intermediate **16** (0.275 g, 0.35 mmol) in THF (3 ml) was added 1 N TBAF (0.46 ml, 0.46 mmol). The mixture was stirred at 25 °C for 1 h and then quenched with ammonium chloride solution (5 ml), the residue was then extracted with ethyl acetate (5 ml), the combined organic layer was washed with brine and dried over Na_2_SO_4_, filtered, then concentrated to obtain crude product which was purified by silica gel chromatography using CH_2_Cl_2_/MeOH (99:1) to afford pure *N*-(3-fluoro-4-((2-(3-hydroxyazetidine-1-carboxamido)pyridin-4-yl)oxy)phenyl)-1–(4-fluorophenyl)-4-methyl-6-oxo-1,6-dihydropyridazine-3-carboxamide as a white solid. Yield: 82.4%; HRMS (ESI, m/z) calculated for C_27_H_23_F_2_N_6_O_5_ (M + H)^+^ 549.1698, found 549.1696.^1^H NMR (600 MHz, DMSO-*d*_6_) δ 10.80 (s, 1H), 9.16 (s, 1H), 8.11 (d, *J* = 5.7 Hz, 1H), 7.90 (dd, *J* = 12.8, 2.0 Hz, 1H), 7.79 (dd, *J* = 8.9, 5.0 Hz, 2H), 7.58 (d, *J* = 8.7 Hz, 1H), 7.48 (d, *J* = 2.0 Hz, 1H), 7.37 (t, *J* = 8.8 Hz, 3H), 7.07 (s, 1H), 6.62 (dd, *J* = 5.7, 2.3 Hz, 1H), 5.60 (d, *J* = 6.4 Hz, 1H), 4.41 − 4.34 (m, 1H), 4.12 (t, *J* = 7.3 Hz, 2H), 3.68 (dd, *J* = 8.3, 3.8 Hz, 2H), 2.42 (s, 3H). ^13^C NMR (151 MHz, DMSO-*d*_6_) δ 165.5, 162.3, 161.7 (d, *J* = 246.1 Hz), 159.0, 156.3, 155.6, 153.8 (d, *J* = 245.6 Hz), 149.6, 143.3, 141.7, 137.5, 137.4 (d, *J* = 3.0 Hz), 136.4 (d, *J* = 12.3 Hz), 130.2, 128.3, 128.3 (d, *J* = 8.8 Hz, 2 C), 117.3, 115.9 (d, *J* = 22.9 Hz, 2 C), 109.2 (d, *J* = 22.7 Hz), 106.4, 98.5, 60.1, 59.7(2 C), 18.7.

### Pharmacology

#### In vitro enzymatic assay

*In vitro* enzymatic activity of **LAH-1** against c-Met was conducted by Mobility shift assay with ATP concentration at Km. Briefly, compound **LAH-1**, MET enzyme (Carna, Cat. No. 08–151), Peptide FAM-P2 (GL Biochem, Cat. No. 112394) and ATP (Sigma, Cat. No. A7699-1G) was diluted with kinase buffer to the designated concentrations. The compound and enzyme solution were added to the assay plate and incubate at room temperature for 10 min. Then the peptide solution was added, incubate at 28 °C for specified period of time, and subsequently added the stop buffer. Data was collected on Calliper, and IC_50_ values were calculated from the inhibition curves.

#### Cell proliferation assays

EBC-1, Hs746T, OE33, SBC-5, U87MG, HT-1080 cell lines were purchased from ATCC, RIKEN, ECACC or JCRB. The antiproliferative activities of **LAH-1** against these cells were assessed by CCK-8 assay according to the manufacturer’s protocol. Briefly, cells were seeded in 96-well plate at different density. 3-fold serial diluted compound or DMSO control was added to each well. After 72 h incubation, CCK-8 was added and incubated for another 2 h. The optical density of each well was read by microplate reader (Biotech, Shanghai, China). IC_50_ values were calculated by GraphPad Prism.

#### Western blotting

Briefly, EBC-1 cells were seeded at a density of 1.5 × 10^6^ cells/well and treated with Compound **LAH-1** at the indicated concentration for 2 h. DMSO-treated cells were used as control groups. The whole cellular protein was extracted and measured by BCA protein assay. Total proteins were fractionated on SDS-PAGE and then transferred onto PVDF membranes (Millipore). Membranes were blocked with 5% BSA at room temperature and then incubated overnight at 4 °C with specific primary antibodies (Phospho-Met (CST, #3077S), Met (D1C2) (CST, #8198S), Phospho-Akt (CST, #4060S), Akt (pan) (C67E7) (CST, #4691S), Phospho-p44/42 MAPK (Erk1/2) (CST, #4370S), p44/42 MAPK (Erk1/2) (137F5) (CST, #4695S)) followed by the secondary antibody. Finally, the detection was performed by the Fully Automated Chemiluminescence/Fluorescence Image Analysis Systems (Tanon 5200).

#### Cell cycle and apoptosis analysis

The effect of **LAH-1** on EBC-1 cells apoptosis was determined by Annexin V-FITC/PI apoptosis detection kit (BD Bioscience, Cat. No. 556547). Briefly, 300,000 EBC-1 cells were seeded in 6-well plate. After treatment with or without **LAH-1** for 24 h, cells were collected, stained by FITC in binding buffer preventing from light, and then labelled by PI (absin, Cat. No. abs50005-50T). The apoptotic cells were analysed by flow cytometer Cytoflex S (Beckman Coulter).

#### Colony formation

EBC-1 cells were trypsinized and approximately 2000 cells were plated on 6-well plates. 14 days later, visible colonies were fixed and stained with 0.1% crystal violet. The number of colonies was quantified by Image J software (Developed by National Institutes of Health). A group of >50 cells was defined as one colony. All experiments were performed in duplicate wells.

#### Cell migration and invasion

NCI-H441 cells in log growth phase were suspended in serum-free medium at a density of 150,000 cells per well and then seeded in 24-well transwell plates (8 μm; Corning). Designated control or **LAH-1** treated cells were added to each migration or invasion chamber and incubated for 28 h. Additionally, HGF (100 ng/mL) was added to the lower well of each companion plate to attract cells from migration or invasion chamber plate inserts at the top of the companion plate. The invaded or migrated cells to the lower wells were then fixed and stained with crystal violet. Images were taken by Olympus BX51 microscope.

#### In vitro ADME assay

The liver microsomal stability assay, PAMPA, and CYP 450 Inhibition Assay were conducted according to the previous reported procedure [19,20].

#### In vivo pharmacokinetics

PK parameters of **LAH-1** was determined in SD rats (male, *n* = 3 per group) weighing (200–240 g), which were obtained from SpePharm (Beijing) Biotechnology Co., Ltd. Briefly, rats were fasted overnight before dosing, **LAH-1** was given as a solution in 10% DMSO with 90% Saline for intravenously (5 mg/kg) and 0.1% PEG400 in 0.5% CMC-Na in water for PO (20 mg/kg). Each rat received 2 or 10 ml of the dosing solution/kg of body weight by intravenous injection and by gavage, respectively. Blood sample was collected at 0.033 (iv only), 0.083, 0.25, 0.5,1, 2, 4, 6, 8, 12 and 24 h after dosing and stored in ice (0–4 °C). Plasma was separated by centrifugation within 15 min post sampling. Levels of **LAH-1** in blood were determined by 6500+ Triple Quad LC-MS/MS System (AB SCIEX LLC, CA, USA). The PK parameters were calculated with WinNonlin software using noncompartmental analysis.

#### Statistical analysis

Data were expressed as the mean ± SD and analysed with GraphPad Prism Software (Version 9.0.0). Statistical comparisons were carried out by one-way analysis of variance (ANOVA) and Student’s t test. Statistical significance was defined as*p < 0.05, **p < 0.01, ***p < 0.001, ****p < 0.0001.

### Molecular docking and dynamics simulation

Molecular docking calculations and simulation were conducted in Yinfo Cloud Platform (http://cloud.yinfotek.com/) as previously reported^20^. The chemical structure of **LAH-1** was converted to 3D structure with energy minimisation in the MMFF94 force field. The c-Met protein coordinate (PDB: 3LQ8) was downloaded from the Protein Data Bank and chosen as templates to compare the docking mode. The redundant atoms were deleted and the protein structure was treated by residue repairing, protonation, and partial charges assignment in AMBER ff14SB force field. The binding pocket was defined according to the original ligand (Foretinib) and spheres were generated. Semi-flexible dockings were performed using the DOCK 6.7 program on the Yinfo Cloud Platform. The Grid-based score was calculated for each pose and clustering analysis was performed to obtain the most reasonable pose. The image files were generated by Pymol. Subsequently, the combined stability of **LAH-1** to c-Met was investigated by Amber 16 software. The ff14SB force field and the general AMBER force field (gaff2) were used for c-Met kinase and **LAH-1**, respectively. The system was solvated by a truncated octahedral box using TIP3P water model with a margin of 15 Å. Sodium ions were added to neutralise the system. The MD simulation protocol comprised the following steps: (1) The limiting potentials of proteins, ligands, and counterions were all restricted by the force constant of 200 kcal/(mol Å), and the energy of the solvent water molecules was minimised to make the water molecules reach a relaxed state. (2) The energy of the system is further minimised. Protein, ligand and ions were subjected to the limiting potential with a force constant of 300 kcal/(molÅ). (3) The restriction potential of the protein backbone was restricted by the force constant of 20 kcal/(mol Å). (4) Then the system was minimised without any restriction. To ensure that the system was in equilibrium, the NPT ensemble was employed to gradually heat the system from 10 k to 300 k at a constant volume over a period of 100 ps. The cpptraj module in AMBER16 was employed for the RMSD calculations.
